# Understanding the Saffron Corm Development—Insights into Histological and Metabolic Aspects

**DOI:** 10.3390/plants13081125

**Published:** 2024-04-17

**Authors:** Claudia Pallotti, Begoña Renau-Morata, Loriana Cardone, Sergio G. Nebauer, Mireia Albiñana Palacios, Alba Rivas-Sendra, José M. Seguí-Simarro, Rosa V. Molina

**Affiliations:** 1 Departamento de Producción Vegetal, Universitat Politècnica de València, Camino de Vera s.n., 46022 Valencia, Spain; pallotti@bvg.upv.es (C.P.); begonya.renau@uv.es (B.R.-M.); sergonne@upv.edu.es (S.G.N.); 2 Instituto Universitario de Conservación y Mejora de la Agrodiversidad Valenciana (COMAV), Universitat Politècnica de València, Camino de Vera s.n., 46022 Valencia, Spain; mireialbinanap@gmail.com (M.A.P.); alba.rivas@basf.com (A.R.-S.); seguisim@btc.upv.es (J.M.S.-S.); 3 Departamento de Biología Vegetal, Universitat de València, C/Doctor Moliner 50, Burjasot, 46100 Valencia, Spain; 4 Department of European and Mediterranean Cultures, Environment, and Cultural Heritage, University of Basilicata, Via Lanera, 20, 75100 Matera, Italy; loriana.cardone@unibas.it

**Keywords:** *Crocus sativus*, meristems, corm growth, vascular connections, carbohydrate metabolism, source–sink relationships

## Abstract

The reproduction of *Crocus sativus* L., a sterile triploid plant, is carried out exclusively through corms, whose size determines the saffron yield. The development of daughter corms (DC) is supported by photoassimilates supplied by the leaves as well as by the mother corms (MC). While biomass partitioning during DC development is well studied, growth dynamics in terms of cell number and size, the involved meristems, as well as carbohydrate partition and allocation, are not yet fully understood. We conducted a comprehensive study into saffron corm growth dynamics at the macroscopic and microscopic levels. Variations in carbohydrate content and enzymatic activities related to sucrose metabolism in sources and sinks were measured. Two key meristems were identified. One is involved in vascular connections between DC and MC. The other is a thickening meristem responsible for DC enlargement. This research explains how the previously described phases of corm growth correlate with variations in cell division, enlargement dynamics, and carbohydrate partitioning among organs. Results also elucidated that the end of DC growth relates to a significant drop in MC root biomass, limiting the water supply for the DC growth, and establishing the onset of leaf wilting. The lack of starch accumulation in aged leaf cells is noteworthy, as is the accumulation of lipids. We hypothesize a signaling role of sugars in DC growth initiation, stop, and leaf aging. Finally, we established a predominant role of sucrose synthase as a sucrolytic enzyme in the maintenance of the high flux of carbon for starch synthesis in DC. Together, the obtained results pave the way for the definition of strategies leading to better control of saffron corm development.

## 1. Introduction

Geophytes have evolved a dual reproductive strategy. These plants develop subterranean vegetative propagation organs, providing an alternative reproductive mechanism. This adaptation ensures their survival and distribution during adverse environmental conditions that may hinder flowering and seed formation [1]. Saffron (*Crocus sativus* L.) belongs to this group of species. However, it is the only sterile triploid species of the *Crocus* genus, and is only propagated vegetatively via its corms [2,3].

In the Mediterranean area, saffron flowering occurs during autumn (October–November) and is followed by a vegetative stage throughout winter, when daughter corms are formed at the base of the shoots. At the beginning of the dry season (April–May), the leaves senesce and wither, and the corms go into dormancy [4]. The transition from the vegetative to the reproductive stage occurs during early summer (July) in the apex of the buds of underground corms [5].

With the aim of controlling or prolonging the flowering period, the stages of the saffron life cycle corresponding to flower induction and sprouting have been studied via anatomical, physiological and transcriptomic approaches [4,5,6,7,8,9,10,11,12,13,14,15]. However, although there is a need to control the size of the daughter corm, given its positive influence on saffron flowering and saffron yield [16,17,18,19,20,21], the literature has paid less attention to the process of corm development.

Different studies indicated that the growth rate of the daughter corms began to increase between late January and early February [19,21]. Daughter corm biomass reached the maximum value in April and was maintained during the initial stage of leaf senescence. However, the correlation of these growth parameters with the processes taking place at a microscopic level is unknown. The duration of the cell division period, the progression of cell size, and whether thickening meristems contribute to the enlargement of daughter corms, or if it is a result of diffuse growth, remain unknown. The timing and way in which vascular connections are established between the mother and daughter corms are also undisclosed. Acquiring such knowledge would improve the design of the research aimed at optimizing daughter corm sizes.

It is also of great interest to gain insights into the metabolic changes inducing corm initiation and the variations during its development, especially those resulting in the arrest of corm growth, the drying up of the leaves, and the dormancy of the meristems. In this respect, geophytes respond to the perception of carbohydrate availability and sugar metabolism, linking the complex sink–source relationships to initiating underground vegetative organ formation or to growth arrest, as has been documented in *Allium cepa* L. [22,23,24] and *Crocus vernus* L. [25].

In saffron, no work has been conducted on addressing the partitioning and allocation of carbohydrates during daughter corm development. The kinetics of starch accumulation in daughter corms, variations in sucrose and hexose content in different organs, and their potential signaling roles remain unknown. Only the metabolic shift in sugars and amino acids during flower primordia development and sprouting, from early May until early October, has been reported [12]. Conversely, corm development is initiated after flowering in November–December and ends at the end of April.

However, investigations into biomass partitioning during the vegetative growth of saffron [19,21] have provided valuable insights into the distinct phases of corm development in relation to the source organs supplying photoassimilates for growth. These studies establish the precise timing of key stages, including growth initiation, leaf aging, and the cessation of daughter corm growth. This chronological framework facilitates the analysis of the involvement of sugars as signaling molecules during these critical stages of corm development.

Renau-Morata et al. [19] showed that corm development initiates post-flowering, and the partitioning of dry matter among different organs shows two distinct phases. In the initial period of vegetative development (end of flowering-mid January), during which the roots and leaves develop, most of the remaining reserves of the mother corm are mobilized to sustain the vegetative growth. The subsequent growth of the daughter corms (end January–April) relies predominantly on leaf photosynthesis and contributes to the largest quantity of the daughter corm dry matter. The growth of daughter corms stops before the full senescence of the leaves, suggesting a sink capacity limitation during their development. When studying *Crocus vernus*, Lundmark et al. [25] found similar results and, according to previous studies by Kock [26], they proposed that the hexose to sucrose ratios control the duration of corm growth in *C. vernus*. A decrease in the ratio of hexoses to sucrose may prompt the shift from cell growth to cell differentiation, reducing the sink strength of the corm, which could be the triggering factor inducing leaf senescence. Similar observations in another geophyte, *Erythronium americanum* Ker Gawl. [27], suggest that as sink limitation intensifies, feedback inhibition of photosynthesis ensues, coupled with the induction of leaf senescence.

Given the scant knowledge regarding carbon partitioning and allocation throughout saffron corm development, it is of major interest to gain insight into this subject during the key stages of corm development: corm initiation, particularly before leaf senescence, and corm growth cessation. This would allow us to elucidate whether a signaling role for sugars, related to the onset and end of corm development, could be considered. Additionally, another important objective is that of unrevealing the enzymes related to carbohydrate metabolism that could be used as targets in breeding programs.

Within this framework and for the purposes of enhancing our understanding of key stages in saffron corm development, this study addresses the following inquiries: (1) How and when are vascular connections established between the mother corm and the newly developing corm? (2) Which specific meristematic tissues are linked to the enlargement of daughter corms? (3) What are the cellular-level growth dynamics of saffron corm and how are they correlated with biomass evolution? (4) How do sugar and starch levels vary throughout corm development, particularly during initiation and late growth stages, and (5) could they play a signaling role? (6) Does leaf aging correlate to significant starch buildup in chloroplasts? (7) Which key enzymes drive carbohydrate metabolism in saffron corms, potentially serving as targets of breeding programs to increase corm size?

To answer these fundamental questions, we have conducted a comprehensive study into the growth dynamics of saffron corms, examining both macroscopic and cellular dimensions. Concurrently, we explored variations in carbohydrate content, and the sucrose-metabolism-related enzyme activities of sources and sinks during this developmental process. Knowing this would facilitate an improvement in the design of the research aimed to optimize daughter corm sizes, and would also enable further molecular-level studies focusing on the identification of the genes regulating vegetative reproduction in this species.

## 2. Material and Methods

### 2.1. Plant Material and Cultivation Conditions

Highly uniform saffron (*Crocus sativus* L.) corms ranging between 26 and 30 g fresh weight (FW) (around 7–8 g dry weight) were used. Corms were purchased from a commercial farm located in Albacete, a traditional saffron production area of Spain. The study was carried out in the experimental fields of the ANECOOP agricultural cooperative located in Museros (Valencia, Spain. 39°34′10.8″ N, 0°21′28.8″ W; 12 msl). Corms were planted in furrows (early September), at a depth of 15 cm, with 15 cm between plants and 50 cm between furrows. Fourteen different blocks, ten plants per block, were randomly distributed in the field. The cultivation practices employed were those commonly used for this crop in Spain [28]. Plants were drip irrigated when rainfed conditions did not meet the water requirements. Temperatures and precipitation in the field are shown in Table S1A.

### 2.2. Sampling and Determination of Biomass Partitioning during the Vegetative Growth Period

In order to study the changes in biomass partitioning during the course of vegetative growth, and especially the processes of corm growth arrest and leaf senescence initiation, the measurements of organ weights were taken on seven dates from the end of the flowering process (last week of November): 2 December, 14 January, 19 February, 24 March, 3 April, 14 April and 23 April. Sampling was performed at a higher frequency in April, the period when the growth arrest of the corm and leaf drying are reported to take place. For each date, 10 plants from two different blocks (5 per block) were sampled. The dry weight of the mother and daughter corms, leaves, and fibrous roots were measured for each plant. In these cultivation conditions, contractile roots did not appear. Plant materials coming from this experiment were also used to analyze cell size and carbohydrate determination.

### 2.3. Processing for Light and Transmission Electron Microscopy

To study changes in size and cell number during corm development, and to identify meristematic tissues, histological sections of saffron corms were observed by means of light microscopy. Corm samples were fixed overnight in FAE (10% formaldehyde, 5% acetic acid, 50% ethanol) at 4 °C, dehydrated in a graded ethanol series, transferred from 100% ethanol to Histoclear gradually, and embedded in paraffin wax (Paraplast Plus; Leica, Nussloch, Germany). Then, 8 μm sections were obtained using a Microm HM 330 microtome (Microm, Walldorf, Germany), mounted onto poly-L-lysine-coated slides and stained with 0.02% toluidine blue. The images were captured by a Nikon Eclipse E600 microscope; Nikon, Melville, USA.

The cell size was determined from corm parenchymatic tissue. The sampling was carried out on the dates previously mentioned (see Section 2.2) from early December to mid-April. To determine the cell size on each date, three samples were used from three different daughter corms, and 1–2 selected slices per corm were studied. From each slice, at least 12 cells were measured along the cross-section by using an eyepiece and stage micrometer. The cross-section surface was determined by taking into account the major and minor diameters. To calculate the cell volume, the cell was assumed to be spheroid in shape. Bearing in mind the cell volume and the volume of the daughter corm (V = 4/3 Πabc; a, b and c are the semi-axes of an ellipsoid), the number of cells per corm was estimated.

To identify the specific meristems involved in the development of the saffron corm together with their time of appearance and activity, a histological study was conducted both when the newly formed corm enters dormancy (late April) and during the early stages of daughter corm development (early December).

In order to clarify whether starch accumulation in chloroplasts takes place during leaf aging, leaves were observed by means of transmission electron microscopy. Leaf samples were fixed in Karnovsky fixative, as described in Satpute et al. [29], and post-fixed with 2% OsO_4_ in 0.05 M cacodylate buffer. The samples were kept overnight at 4 °C in 1% paraformaldehyde in 0.025 M cacodylate, cut into small chunks, and then kept in 0.025 M cacodylate until use. They were dehydrated in a progressive methanol series and embedded and polymerized in Spurr resin (Electron Microscopy Sciences). Ultrathin (80 nm) sections were obtained from embedded samples with a Leica UC6 ultramicrotome. The sections were mounted on carbon and formvar-coated, 200-mesh nickel grids (Electron Microscopy Sciences; Hatfield, USA) and stained with uranyl acetate in 70% methanol (6 min) and lead citrate (30 s). The images were obtained with a Jeol JEM 1010 transmission electron microscope; Jeol, Peabody, USA.

### 2.4. Determination of Carbohydrates

The total soluble sugar and starch levels of the mother corm, the daughter corms, and the leaves were determined on the same seven dates previously mentioned (Section 2.2). For each date, the carbohydrate content was determined from three independent samples. Two different corms were used to obtain each sample. The carbohydrate determinations were performed as described by Ruiz and Guardiola [30]. Briefly, the total soluble sugars and starch were extracted with 80% ethanol and 35% perchloric acid, respectively, and measured using the anthrone reagent [31]. The results were expressed as mg per g of dry weight (mg/g DW).

In order to provide a more accurate evaluation of the involvement of sugars in the processes related to corm growth arrest and leaf senescence, the sucrose, glucose, and fructose contents were determined in the leaves and daughter corms on three different dates: when the corm was actively growing (30 January), shortly before corm growth arrest and after the initiation of leaf senescence (27 March), and shortly after corm arrest and when, at least, half of the leaf was yellowing (24 April). In total, 3 samples of leaves/daughter corms from 2–3 different corms in each sample were harvested on each date. The samples were ground and frozen in liquid nitrogen. The determination of the sugars was performed as described in Minebois et al. [32] by means of gas chromatography (Agilent 6890N, Agilent; Santa Clara, CA, USA) coupled to mass spectrometry (LECO Pegasus 4D TOF, LECO, St. Joseph, MI, USA) at the Instituto de Biología Molecular y Celular de Plantas (UPV-CSIC, Valencia, Spain) Metabolomics Platform.

### 2.5. Enzyme Assays

Considering that harvested organs must be processed immediately for enzymatic determination [33], the enzyme assays were conducted using plants growing in the greenhouse, with 400–1000 μmol m^−2^ s^−1^ irradiance during the day. Climatic values are shown in Table S1B. Approximately, 75 saffron corms were planted in 20 cm deep trays with universal substrate (50%) and coconut fiber (50%) at a planting depth of about 10 cm. The plants were manually fertilized with Hoagland solution No. 2 every 10 days [34]. The samples of daughter corms, mother corm, and leaves (Figure S1) were harvested on three different dates: when the corm was actively growing (17 January), before corm growth arrest (21 March), and shortly after corm growth arrest and once leaf senescence had been initiated (13 April). In total, samples of the different organs from 3–4 different corms in each sample were harvested on each date. The samples were ground and frozen in liquid nitrogen.

In total, 1 g of frozen powder was resuspended at 4 °C in 5 mL of 100 mM HEPES (pH 7.5), 2 mM EDTA and 5 mM dithiothreitol. The suspension was desalted (IVSS Vivaspin 500, Sartorius Biolab, Göttingen, Germany) following the manufacturer’s instructions and assayed for enzymatic activity. The ADPG pyrophosphorylase (AGPP, EC 2.4.1.18), UDPG pyrophosphorylase (UGPP, EC 2.7.7.9), sucrose synthase (SuSy, EC 2.4.1.13) and acid invertase (INV, EC 3.2.1.26) activities were assayed as previously described [33,35,36]. For the detection of the AGPP and UGPP activities, the production of glucose-1-phosphate from ADP-glucose and UDP-glucose was determined, respectively, in an NAD-linked glucose-6-phosphate dehydrogenase system [37]. The NAD reduction was measured spectrophotometrically at 340 nm. The sucrose synthase and invertase activities were measured in the sucrose breakdown direction. The fructose content was determined spectrophotometrically at 340 nm by the NAD-linked hexokinase/phosphoglucoisomerase/glucose-6-phosphate dehydrogenase coupling method. All the enzymatic reactions took place at 37 °C. One unit (U) is defined as the amount of enzyme that catalyzes the production of 1 mmol of product per min.

### 2.6. Statistical Analysis

The data from different treatments were compared and analyzed by using a factorial or one-way ANOVA (Statgraphics Centurion XVIII for Windows, Statgraphics Technologies, Inc., The Plains, VA, USA). The means were compared at a 5% level of probability using the LSD multiple range test.

Figure S2 presents a concise diagram summarizing the analyzed characters and corresponding sampling dates across the different experiments conducted in this study.

## 3. Results

### 3.1. Meristems Specifically Involved in the Development of the Saffron Corm

**Figure 1:**
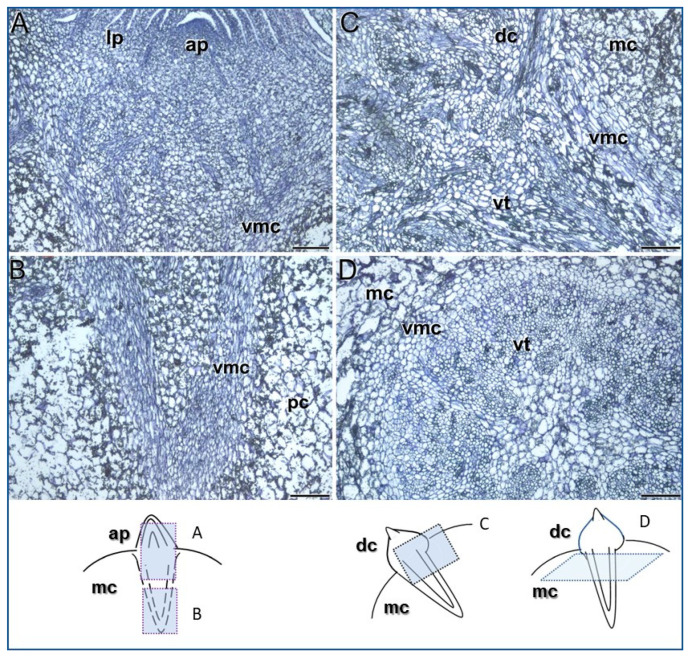
Vascular connection between the mother and the daughter corms. (**A**,**B**) Longitudinal section of the dormant mother corm, at the end of April, showing the apical bud that will give rise to the daughter corm (**A**), as well as the wedge-shaped meristematic tissue that will develop the vascular connection between the mother and the daughter corms (**B**). (**C**,**D**) Longitudinal (**C**) and transverse sections (**D**) of the daughter corm in development showing vascular connection between the mother and the daughter corms, as well as the meristematic cells producing them. ap, apical meristem; lp, leaf primordia; vmc, meristematic cells that will give rise to vascular tissues; pc, parenchyma cells; dc, daughter corm; mc, mother corm; vt, vascular tissues. Bars: 200 µm.

Histological analysis revealed that in the mother corm, after leaf senescence and the onset of dormancy at the end of April, a wedge-shaped meristematic tissue is already formed, just below the bud that will give rise to the new daughter corm (Figure 1A,B). This boundary meristem displays several layers of elongated cells (Figure 1B). As the daughter corm begins to grow, multiple vascular elements differentiate in a conical distribution connecting both daughter and mother corms (Figure 1C,D). Furthermore, the outermost layers of the structure may become an abscission zone when the vascular connection between mother and daughter corms breaks down by the end of the vegetative cycle.

**Figure 2:**
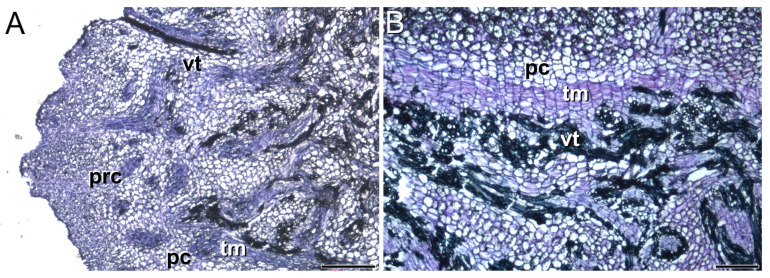
Initial development of the underground stem (early December). (**A**) Longitudinal section of the developing corm showing vascular tissues coming from the procambium (near the apex), as well as from a thickening meristem (below the apex). (**B**) Detail of thickening meristem producing primary vascular tissue and parenchyma cells. prc, procambium; vt, vascular tissues; pc, parenchyma cells; tm, thickening meristem. Bars: 200 µm.

The thickened underground stem (the future daughter corm) initiated growth in December, after flowering. Disjunct strands of elongated meristematic cells, differentiating into vascular tissues, were observed in accordance with the scattered nature of vascular bundles in monocots (Figure 2A). Near the apex, these vascular tissues may be derived from procambial cells. However, in the region below the apex, discrete patches of tangentially flattened cells could have given rise to vascular and ground tissue (Figure 2B), resembling a thickening meristem. The activity of these meristematic cells contributed to the increase in thickness of the daughter corm, which, by mid-January, was clearly distinguishable in the apical part of the mother corm.

Together, our data reveal that two distinct meristem types must play a pivotal role in the development of the daughter corm: one seems to be responsible for establishing vascular connections between the mother and the daughter corms, while the other is involved in the thickening of this modified monocot stem.

### 3.2. Growth Dynamics of Saffron Corm at Macroscopic and Cellular Levels, and Its Relationship with Biomass Changes in the Source Organs

**Figure 3:**
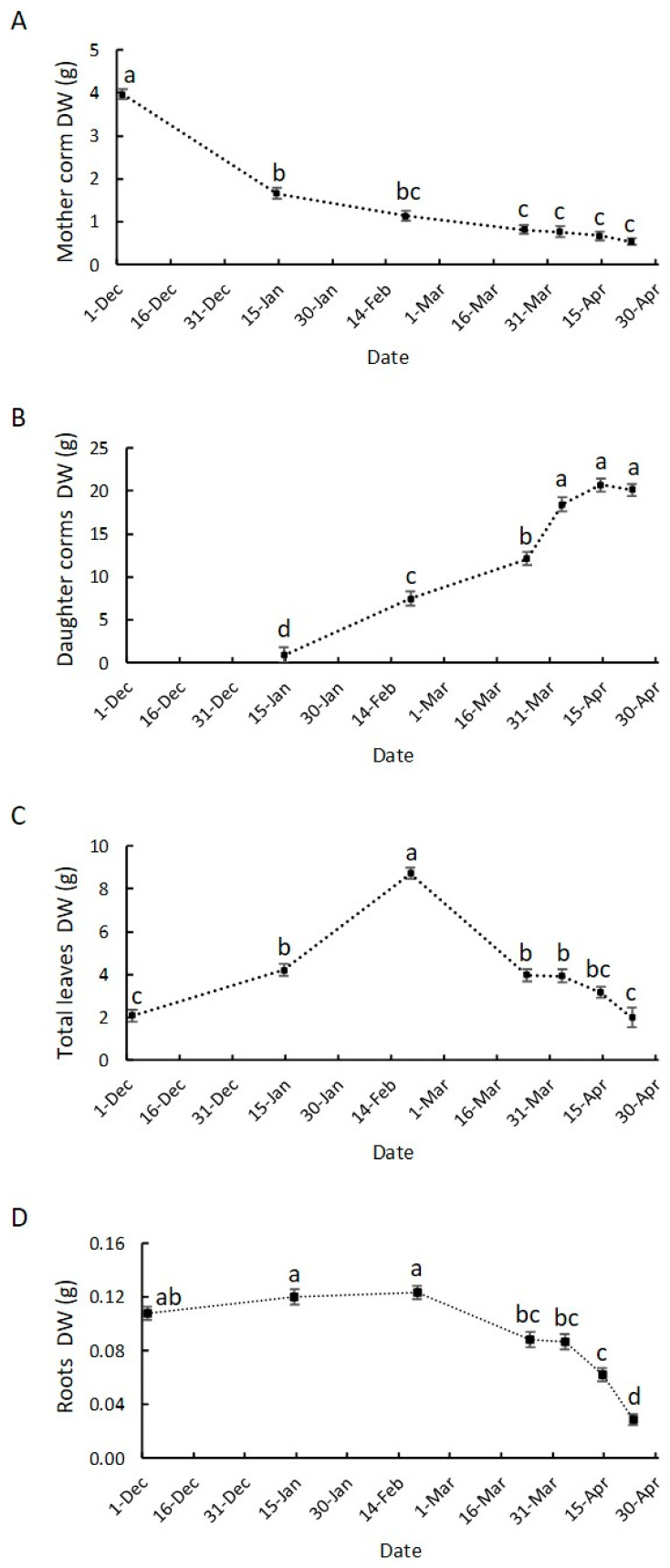
Time course of mother corm dry weight (**A**), daughter corm dry weight (**B**), total leaf dry weight (**C**) and root dry weight (**D**) (from 2 December to 23 April). Data are the means ± SE of 10 independent replicates. Different letters indicate significant differences between dates (*p* < 0.05).

The measurable growth, as detachable structures, of the daughter corms began in mid-January (Figure 3B). The growth rate was maintained until the second half of March. The maximum value of daughter corm biomass was reached in April and maintained during the final stage of development characterized by the lignification of the cataphylls.

**Figure 4:**
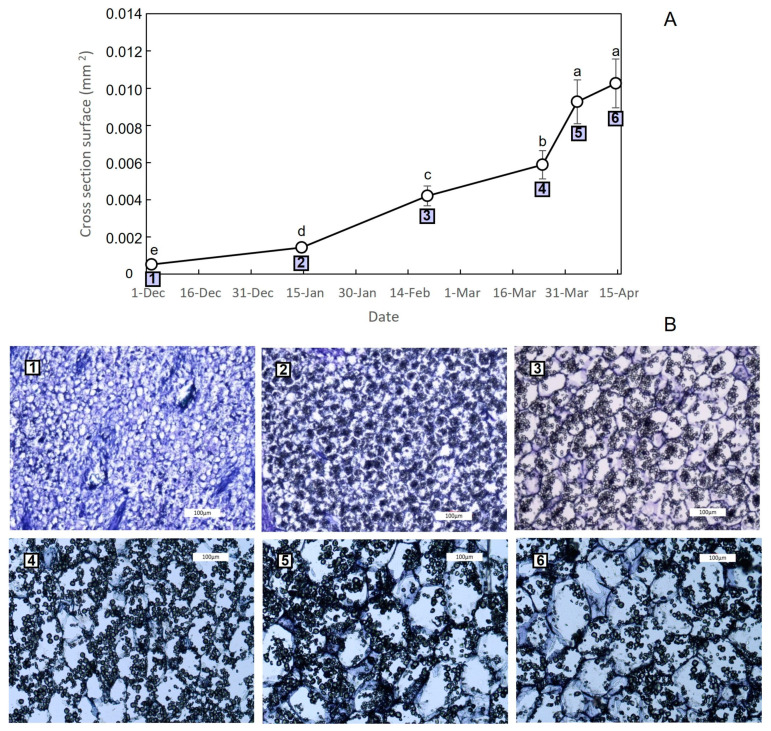
Increase in cell size (cross-section surface) during saffron daughter corm development. For each date, at least 40 cells were measured from 3 different slices. Different letters indicate significant differences between dates (*p* < 0.05). (**A**) Increase in cross-section surface (mm^2^) over time. (**B**) Representative image of the increase in cell size. Numbers relate to cross-section surface and cell size images on the same date. (**1**) 2 December; (**2**) 14 January; (**3**) 19 February; (**4**) 24 March; (**5**) 3 April; (**6**) 14 April. Bars: 100 μm.

At the microscopic level, a 6-fold increase in the number of daughter corm cells was observed from mid-January, when corms were apparent, to mid-February (from 7.6 to 42 million cells. Figure S3). However, from this date onwards, the number of cells increased to a much lesser extent (from 42 to 51 million cells in early April), with the increase in daughter corm biomass mainly influenced by an enlargement in cell size. The increase in cell size could be noted from December to early April (Figure 4) and exhibited a close correlation (r = 0.97. *p* < 0.05) with the increase in corm dry weight. Notably, there is a marked similarity between the biomass growth pattern (Figure 3B) and the pattern corresponding to the increase in cell size (Figure 4).

The variations in the weight of the other organs reflect the source–sink relationships during vegetative development in this species. When the growth of the daughter corm became apparent, 50% of the mother corm reserves were depleted (Figure 3A), the roots had already reached their maximum development, and the leaves had already started to grow but their main development occurred afterward in mid-February (Figure 3C,D).

Between late March and early April, when the daughter corm attains maximum growth, the mother corm reserves are almost entirely depleted, and the roots developing from it indicate an increased rate of dry weight reduction from this time onwards, as do the leaves (Figure 3). However, the growth of the replacement corms ceases prior to the total senescence of leaves and roots (Figure 3A–D).

### 3.3. Changes in Carbohydrate Partition and Allocation during Corm Development

**Figure 5:**
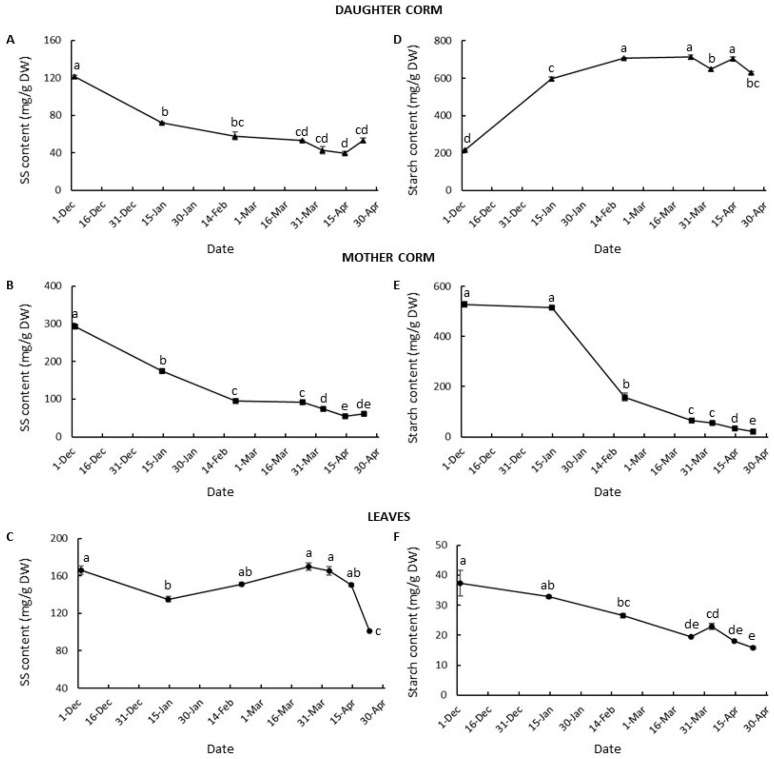
Changes in total metabolizable carbohydrates, soluble sugars [SS] (**A**–**C**), and starch (**D**–**F**) in daughter corms (**A**,**D**), mother corm (**B**,**E**) and leaves (**C**,**F**) of saffron, during the vegetative period. Values are averages of three independent samples ± SE. Within each graph, different letters indicate significant differences (*p* < 0.05) between dates.

The dynamic changes in carbohydrates in the plant during daughter corm development showed a photoassimilate partition primarily aimed at supplying the demands of the daughter corms, as the main sinks in the plant (Figure 5).

In the daughter corm, the total soluble sugar content was high in early December, coinciding with the early swelling period, and it declined rapidly until mid-February when the number of cells was no longer increasing (Figure 5A and Figure S3). From this point on, the total soluble sugar content remained almost constant, at around 40–50 mg/g of DW. By contrast, rapid synthesis and accumulation of starch were produced from early December until the second half of February, when the starch content reached the maximum value, of around 700 mg/g DW (Figure 5D). From this point onwards, the starch content remained stable, despite the continued increase in cell size.

In the mother corm, in accordance with its role as a source organ, a steady decrease in the content of total soluble sugars and starch was observed (Figure 5B,E). However, the patterns were somewhat different. The high level of total soluble sugars (292 mg/g DW) noted in early December decreased sharply in line with the growth of the roots and leaves, and the initial swelling of the daughter corm, until it reached 95 mg/g DW in the latter part of February (Figure 5B). From this date on, the soluble sugar content changed slightly, reaching its lowest value in mid-April. However, the starch content did not decline until mid-January (Figure 5E), when the growth rate of the daughter corm increased (Figure 3B). One month after that date, a decrease in starch content of more than 70% was noted in the mother corm (Figure 5E). Thus, in the second half of February, the starch content had declined from 514 to 158 mg/g of DW. At this time, the maximum growth of leaves and roots, and the maximum daughter corm starch content had been reached. The daughter corm had achieved 38% of its final dry weight at this point. From this point onwards, the starch content in the mother corm continued to decline gradually until the end of the cycle. It is interesting to note that while in the daughter corm there was a negative correlation between the variation in the content of total soluble sugars and starch (r = −0.94; *p* < 0.05), in the mother corm, the changes in these parameters exhibited a positive correlation (r = 0.92; *p* < 0.05).

In the leaves, the total soluble sugar levels remained fairly stable until the end of April, when a significant drop in soluble sugar content took place shortly before the leaf was totally dry (Figure 5C). Nevertheless, there was a steady decline in the starch content from December until the end of April (Figure 5F). However, it is necessary to point out that there was a slight increase in starch levels in early April when the daughter corms reached the maximum dry weight.

By analyzing the absolute values of the soluble sugar and starch contents in the different organs, and their partitioning over time, we observed that before mid-January most of the starch was found in the mother corm (Figure S4), but already in mid-February more than 90% of the total starch was found in the daughter corm. The starch content of the leaves was at best 8% of the total starch content. The highest proportion of the total soluble sugars was found in the leaves between mid-January and the end of March. From this point onwards, more than 50% and up to 82% of the total sugars were found in the daughter corm.

### 3.4. Corm Growth Arrest and Leaf Senescence in Saffron: Relationship with Hexose to Sucrose Ratios

**Figure 6:**
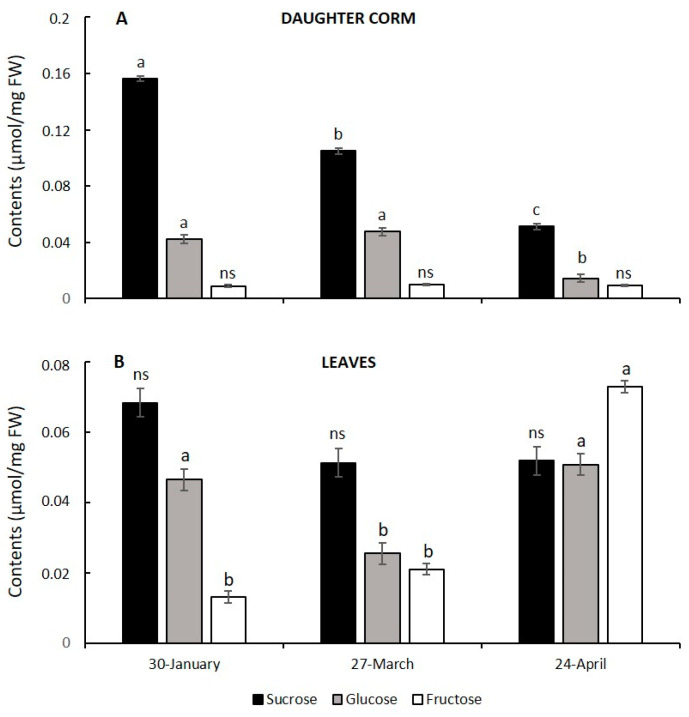
Changes in sucrose, glucose and fructose in daughter corms (**A**) and leaves (**B**) of saffron, on three different dates throughout the vegetative period: when the corm is actively growing (30 January), shortly before corm growth arrest and after the initiation of leaf senescence (27 March), shortly after corm arrest and when, at least, half of the leaf is yellowing (24 April). Values are averages of three independent samples ± SE. In each graph, different letters indicate significant differences (*p* < 0.05) between dates for each sugar. ns indicates no significant differences.

**Figure 7:**
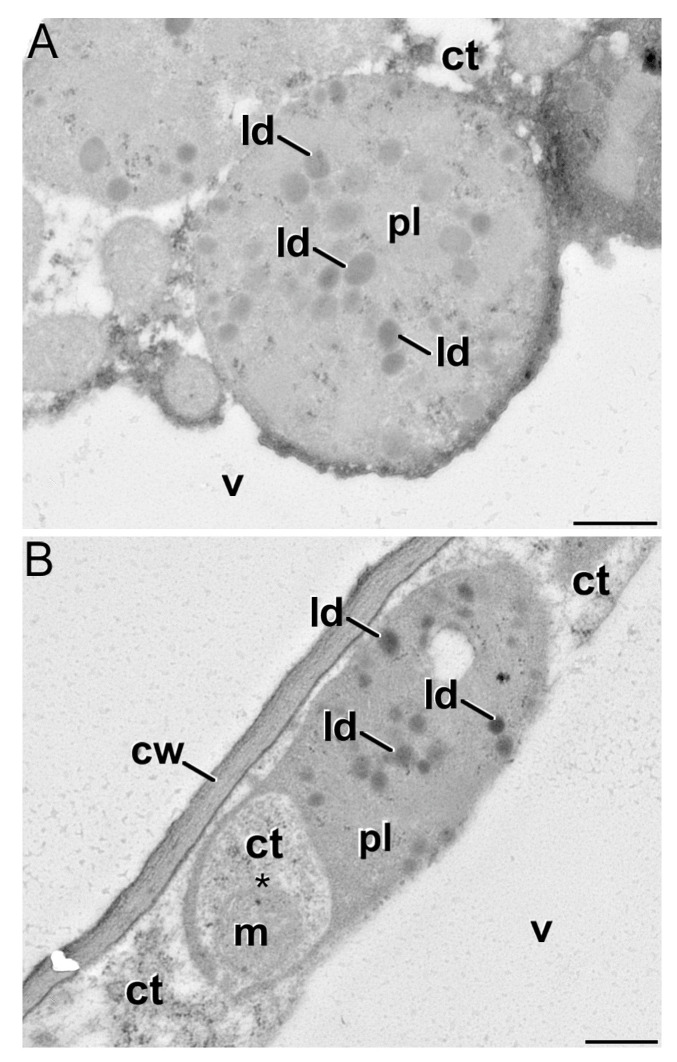
Transmission electron microscopy of plastids of aged, yellow leaf cells showing abundant lipid deposits (ld) (**A**). In (**B**), note the occurrence of a plastid (pl) undergoing intraplastidial autophagy, whereby a cytoplasmic region (asterisk) including a mitochondrion (m) is being engulfed by the plastid (plastolysome). ct: cytoplasm; cw: cell wall; v: vacuole. Bars: 500 nm.

The proposal has been put forward that hexose to sucrose ratios might control the duration of corm growth in some spring crocuses, and that leaf senescence may be induced by a reduction in daughter corm carbohydrate demand [25]. In order to determine whether specific changes in sugar contents control corm growth duration in saffron, we quantified sucrose and hexose (glucose and fructose) contents at key developmental stages of corm development. Our results demonstrated that the corm sucrose content dropped during corm development (Figure 6A). Of all the measured sugars, the highest concentration was that of sucrose, which also underwent the most dramatic changes over time, reaching only 33% of its initial value by the end of corm growth. The glucose content in the corm decreased, but only at the end of the cycle; fructose, on the other hand, did not exhibit any significant changes over time, its concentration being the lowest. Thus, the ratios of hexoses to sucrose did not decrease, but rather increased, mainly due to the decline in sucrose content. By contrast, leaf sucrose contents did not change significantly over time. Nevertheless, the highest levels of hexoses were reached towards the end of the cycle (Figure 6B), with fructose showing the highest concentration at this time. There was no significant accumulation of starch found in the leaves towards the end of the corm development (Figure 5F and Figure 7). However, transmission electron microscopy showed a noteworthy accumulation of lipids in plastids when the leaves turned yellow (Figure 7). This was paralleled by the occurrence of intraplastidial autophagy, a process whereby some plastids invaginate their double-membrane envelopes and engulf portions of the cytoplasm (Figure 7B), generating a cytoplasmic compartment within the plastid, isolated from the outer cytoplasm [38]. Then, plastids become lytic compartments (plastolysomes), digesting themselves as well as their cytoplasmic cargo. Intraplastidial autophagy has been related to senescence in several plant tissues [39]. Thus, the fact that this process occurs represents added evidence of leaf senescence at this moment.

### 3.5. Activities of Enzymes Involved in the Metabolisms of Sugar and Starch

With the aim of finding enzymes that could be a target for breeding work to increase the size of the saffron corm, we measured the activities of different enzymes involved in the metabolism of sucrose and starch: ADP-glucose pyrophosphorylase (AGPPase), which controls the key regulatory step of starch synthesis, UDP-glucose pyrophosphorylase (UGPPase), invertase (INV) and sucrose synthase (Susy), regulating the sucrose metabolism [35].

**Figure 8:**
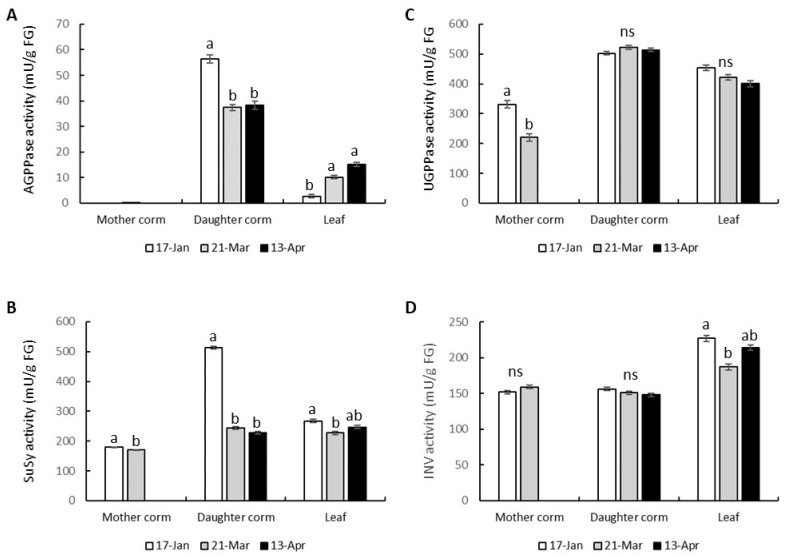
Changes in the activity of AGPPase (**A**), Susy (**B**), UGPPase (**C**), and INV (**D**) in the mother corm, daughter corm, and leaves, through three different stages of the vegetative growing season: when the corm is actively growing (17 January), before corm growth arrest (21 March), and shortly after corm growth arrest and once leaf senescence has been initiated (13 April). Values are means ± SE of 3 determinations, each coming from 3–4 different plants. In each graph, different letters indicate significant differences (*p* < 0.05) between dates in each organ. ns indicates no significant differences.

A peak of AGPPase activity appeared in the daughter corm around mid-January (Figure 8A), when a marked increase in starch content took place (Figure 5D). At that time, the partitioning of the total starch content to the daughter corm increased sharply. The largest increase in the percentage of starch in the daughter corm took place from mid-January until the second half of February (Figure S4). The total starch increased by more than 900%. The AGPPase activity fell by 34% between the end of March and mid-April. Over this period, the total starch content of the daughter corm did increase, but to a smaller extent (68%). The AGPPase activity in the mother corm was negligible, in accordance with its role as an irreversible source organ during the entire cycle (Figure 3 and Figure 8A). TheAGPPase activity in the leaf was much lower than in the daughter corm; this activity, however, increased towards the end of the cycle, before leaf senescence. A slight rise in the starch content of the leaf is observed in early April (Figure 5F). However, there is no significant accumulation of starch in the leaf at the end of the cycle (Figure 7).

Our results (Figure 8A,B) also confirmed a relationship between Susy and AGPPase activity in the starch-storing daughter corm. The greater quantity of hexoses required for starch synthesis purposes during mid-January, as indicated by the AGPPase activity, seems to be supplied by the product of the Susy activity which was also more active during that time. There were no significant differences in the INV activity (Figure 8D) of the diverse samples taken throughout the life cycle of the daughter corm.

The overall activity of UGPPase (Figure 8C), which catalyzes a reversible reaction and converts UDP-Glucose and PPi into glucose-1-phosphate and UTP, did not change during the daughter corm development. The only significant decline in its activity was related to the decrease in the availability of carbohydrates to be exported into the mother corm.

## 4. Discussion

### 4.1. Two Distinct Types of Meristems Play a Pivotal Role in Saffron Corm Growth

Our results have shown that upon initiation of saffron daughter corm development in early December, the establishment of vascular connections between the emerging shoot and the mother corm is already underway. Vascular tissues are developed from meristematic cell layers arranged in a wedge-shaped configuration at the interface of both structures. The establishment of this connection is essential as the developing daughter corm lacks roots, and thus, water and nutrients are provided through the mother corm. The presence of numerous vascular connections linking the mother and the replacement corms has previously been described in saffron [4]. However, their formation from pre-existing meristematic cells and a pre-existing abscission zone-like structure between the old and new corms had not been observed. Interestingly, this meristem can be observed from the onset of dormancy at the end of April.

In monocotyledons, both the apical meristem and the primary thickening meristem develop the primary body by means of a gradual increase in thickness [40,41,42]. The increase in underground organ thickness in the *Iridaceae* family has mainly been studied in species that develop bulbs [43]. Corm-forming species have received less attention. The analysis by Yasui et al. [44,45], studying Freesia (*Freesia hybrida* cv. Ryneveld’s Golden Yellow) and Gladiolus (*Gladiolus grandiflora* hort. Cv. Traveller.), pointed out that the increase in thickening in these species was caused by actively dividing cells which diffused in permanent tissues of daughter corms. These dividing cells did not form any meristematic tissue. However, our results show that the growth of the saffron daughter corm arises from the activity of the apical meristem but that, additionally, in the region below the apex, discrete patches of tangentially flattened cells give rise to vascular and ground tissue, resembling a thickening meristem.

Taken together, these results renew interest in this field; this is important for a more comprehensive understanding not only of the thickening of saffron corm, but also of the abnormal thickening growth in monocotyledons with corms.

### 4.2. The Growth of the Saffron Corm as a Set of Coordinate Processes including Cell Division, Cell Expansion, as Well as Changes in Carbohydrate Partitioning and Allocation

Resembling what occurs in rhizomes or tubers [46,47], the swelling of the corm involves a set of processes that includes cell division, cell expansion, and complex physiological changes including variations in biomass, carbohydrate content and related sucrose and starch metabolisms [48]. To our knowledge, this is the first research work into the physiology of saffron corm development that focuses on the metabolisms of sugars and starch throughout this process that also includes histological aspects related to the growth dynamics.

Regarding this study, the changes in biomass partitioning among organs during the growth cycle are similar to those previously observed [19,21] showing two distinct phases.

From early December to mid-February, the supply of carbohydrates from the mother to the daughter corm will allow the initial swelling stage by increasing the cell number and cell size; at the same time, a high starch content is reached in these cells, which are still growing. During this period, a clearly visible increase in daughter corm size takes place. From this time onwards, the relevance of the mother corms as a source of carbohydrates declines, as has previously been described in saffron and other *Crocus* species [19,21,25]. From mid-February, there was a slow and slight decrease in the content of soluble sugars and starch in the mother corm, which will reach its minimum in mid-April. However, it is important to note that the mother corm is not fully consumed until the end of the cycle. It allows the maintenance of the roots, which supply water, minerals and other compounds, such as hormones, to the rest of the plant.

By mid-February, the capacity of the leaves to act as a source organ is at a maximum, and from this time onwards, the growth of the daughter corm relies on the photosynthetic activity of the leaves. At this time, cell divisions have mostly ceased, but cell growth continues until the beginning of April, while there is a sustained high starch content in the corm cells. The great sink strength of the daughter corm will lead to a steady decline in the starch content of the leaf. However, the total soluble sugar levels related to dry weight remain fairly stable until shortly before the leaf is totally dry. This constancy could be explained by the close coordination of source organ photosynthetic activity with the carbon demand of sink organs, pointing to a decrease in the photosynthetic rate when sink demand for carbohydrates is limited [49,50]. The decrease in the sink strength of the daughter corm in early April leads to a transient rise in the starch content of the leaf. However, no significant accumulation of starch in the leaf at the end of the cycle has been observed. Instead, large droplets containing a lipid-like substance accumulate in the chloroplast, as has been described in yellowing leaves [51]. A high lipid content in saffron leaves has also been described by Jadouali et al. [52] when studying the composition of saffron by-products in relation to their use as animal feed. As noted by the authors, further study into the lipid content in saffron leaves may be of interest for the purposes of enabling farmers to diversify their sources of income, such as feed for livestock, and to make the saffron market more profitable.

The change in the total soluble sugar content in the daughter corm is in accordance with the role of these compounds as sources of energy and carbon skeletons for the purposes of increasing cell number and size, as well as for starch accumulation. The starch content was negatively related to the soluble sugar content, since the process of corm development is the result of soluble sugar consumption and starch accumulation via the action of a series of related enzymes, as pointed out by Miao et al. [48] in *Tulipa edulis*. However, the accumulation pattern through development may vary from species to species. In saffron, the starch content rises sharply from early December onwards and reaches its highest level in February, when the cell size is only 36% of the final size. This pattern differs from that observed in other underground organs, such as freesia corm [53] or *Tulipa edulis* stolon [48]. In such species, starch synthesis and accumulation occur in the middle and later stages.

### 4.3. Sugars as Signaling Molecules for Corm Initiation, Corm Growth Arrest and Leaf Senescence

Although the main role of sugars in metabolism is as carriers of energy and carbon, they play several additional roles, such as maintaining osmotic potential and signaling the energy status of plant parts [51]. The high levels of soluble sugars observed in December might be a requirement for storage organ initiation in saffron. Fernie and Willmitzer [46] discussed how soluble sugars, most notably sucrose, have convincingly been considered strong inducers for the formation of underground storage organs. In addition, the work of Cheng et al. [54] on *Sagittaria trifolia* showed that enhancing the levels of sucrose is helpful for corm formation.

Our results confirmed sucrose as a major soluble sugar in the daughter corm, reaching its peak concentration during the phase of cell divisions and gradually decreasing as corm growth stops.

The role of sugars as signaling molecules in the cessation of corm growth and leaf senescence has also been put forward. Lundmark et al. [25] proposed that hexose to sucrose ratios might control the duration of corm growth in *Crocus vernus* by influencing the timing of cell division, elongation, and maturation phases. The faster the decrease in the ratio of hexose to sucrose takes place, the sooner the elongation period will end, and the greater the reduction in the final biomass of the corm.

Our data showed that there was no decrease in the hexose/sucrose ratio either when the cells had already ceased to divide significantly or when they stopped increasing in size. However, prior to the cessation of cell growth, when the starch and sugar content of the mother corm is already low, a decrease in root biomass may be observed that closely matches that of the leaves, and which is more marked at the beginning of April when the growth of the daughter corm cells ends. This suggests that the cessation of growth could firstly be related to a decrease in the supply of water via the roots. Although a decrease in the supply of photoassimilates through the leaves may be ongoing from the end of March onwards, the leaves are still largely green when the daughter corm stops growing. Taking into account the significant decrease in the soluble sugar content (mainly sucrose) of the daughter corm during its development, we cannot rule out the signaling role of sugars through the regulatory network that links growth to changes in the nutrient and energy status by means of the plant Snf1-related kinase 1(SnRK1), and the plant target of rapamycin (TOR) kinase [55]. However, the relationship between the expression of the above-mentioned genes and the level of sugars requires further research work at the molecular level.

A decrease in the sink strength of the corm could be a factor in the induction of leaf senescence, and this process could be mediated by a rising sugar level, as reviewed by van Doorn [51] and as has been pointed out in studies into *C. vernus* [25]. However, prior to that point, and as has previously been mentioned, there has already been a reduction in the root system that can reduce the flow of cytokinins into the leaf. Thus, a slight increase in the sugar and starch content in April and, prior to that, a decrease in the supply of cytokinins from the roots, could trigger leaf senescence. It is noteworthy that, during the leaf aging phase, there is no significant accumulation of starch in the chloroplasts that would initiate starch-induced leaf chloroplast disintegration [56]. It is also interesting to point out that the soluble sugar that increased the most in the yellowing leaf is fructose.

### 4.4. There Is a Predominant Role of Susy as a Sucrolytic Enzyme in the Maintenance of a High Flux of Phosphorylated Glucose Directed at Starch Accumulation in the Daughter Corm

The study of the enzymes involved in a metabolic pathway and the changes in their activity may be of great interest for the purposes of defining targets for yield improvement. With this objective in mind, we studied the activity of the different enzymes involved in carbohydrate metabolism throughout the development of the saffron corm. The most noteworthy findings were observed for AGPPase, which controls the synthesis of starch by catalyzing the formation of adenosine diphosphate glucose (the precursor of starch), and for Susy, involved in the cleavage of sucrose.

The inter-organ variations in both the activity of AGPPase and also during plant development correlated well with the changes in the starch content, as has been observed in the growth of other underground vegetative propagation organs, such as tubers, stolons or other corms [46,48,53,57]. In other ornamental species propagated by corms, such as gladiolus, the *AGPPase* gene has been cloned and characterized at the molecular level [57], and it has been proposed that the quantity and quality of gladiolus corms could be improved by enhancing the *GhAGPS1* gene expression through genetic engineering techniques. It is also interesting to point out the role of GAs in the regulation of *AGPPase* expression in some bulbous plants [5,58].

As sucrose is the major photoassimilate form delivered to the daughter corm, and the starting point of starch synthesis in the development of vegetative storage organs, the mechanisms responsible for the breakdown of sucrose, and their regulation, are of particular interest [59]. One important question concerns the relative roles of the two different types of sucrolytic enzymes [60,61]. Sucrose can either be hydrolyzed by invertase, resulting in glucose and fructose, or converted into UDP-glucose (UDP-Glc) and fructose by sucrose synthase. It has been well established in different species with subterranean storage organs that, in their starch-storing sinks, incoming sucrose is degraded predominantly via the Susy pathway [46,48,60,62,63,64]. Also in saffron, and based on the activity patterns in the daughter corm of the sucrolytic enzymes, it seems that Susy constitutes the predominant route for sucrose breakdown if a high flux of carbon is to be maintained for growth and storage in the corm. The enhancement of Susy’s activity represents a useful strategy for increasing starch accumulation and yield in potato tubers [63].

**Figure 9:**
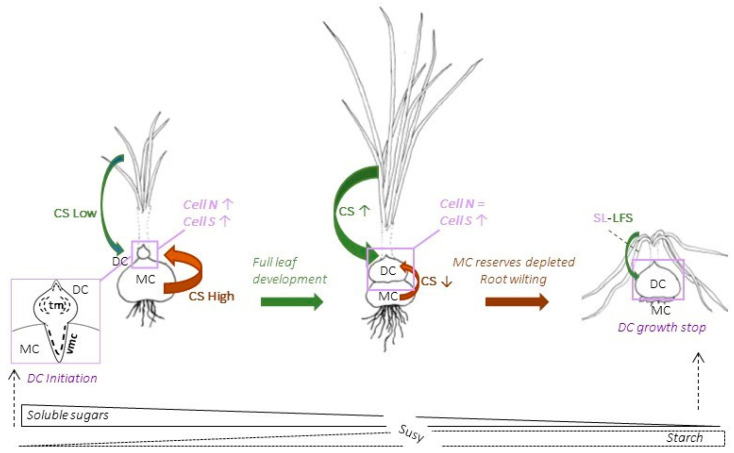
Saffron corm development. See the main text for explanation. Abbreviations and symbols: DC, daughter corm; MC, mother corm; vmc, meristematic cells that will give rise to vascular tissues; tm, thickening meristem; Cell N, number of cells; Cell S, cell size; CS, Significance as a carbohydrate source organ; SL-LFS, sink limitation-leaves achieving full senescence. ↑, parameter increasing; ↓, parameter decreasing; =, unchanging parameter.

In summary, the development of the daughter corm in saffron is initiated not only by establishing vascular connections with the mother corm, allowing the transport of water and nutrients, but also via the establishment of the mother corm as a source organ. In an early stage, the daughter corm increases the number of cells and their size, and reaches maximum starch concentration, while the content of soluble sugars, which is very high at the beginning of its development, decreases. In this stage, the leaves, although not fully developed, can also function as a source organ (Figure 9). In the second stage, the corm will gain weight due to an increase in cell size, maintaining the high concentration of starch reached in the first stage. The source organs are the leaves, in which the soluble sugar content remains constant despite the reduction in the starch reserve. The cessation in the growth of the corm could be related to a significant drop in root biomass that would limit water supply and initiate leaf aging. The variations in sugar levels as a consequence of these processes could signal the end of corm growth and leaf senescence (Figure 9).

## 5. Conclusions

In conclusion, this study has significantly increased our understanding of various aspects of saffron corm development. We have identified two key meristems in this process, elucidating the origin of the vascular connections between the daughter and the mother corms, as well as a thickening meristem responsible for the daughter corm enlargement. This research has explained how distinct phases of corm growth correlate with variations in the dynamics of cell division and enlargement, as well as with carbohydrate partitioning among different organs. Furthermore, we have elucidated the physiological processes involved in the cessation of daughter corm growth, highlighting the possible mediating role of sugars as signaling molecules in both the initiation and termination of corm growth, as well as in leaf aging. All of this knowledge will enable us to enhance the design of research aimed at optimizing corm size through environmental or hormonal factors. Additionally, it will facilitate the investigation of key genes that integrate environmental responses and carbon status to promote vegetative reproduction [1]. Moreover, we have identified specific enzymes that could serve as targets for the purposes of increasing corm growth either via conventional or biotechnological approaches. Finally, the results about lipid content in saffron leaves enabling farmers to diversify their sources of income should not be underestimated.

## Data Availability

All related data are available within the manuscript and Supplementary Materials.

## Supplementary Materials

The following supporting information can be downloaded at: https://www.mdpi.com/article/10.3390/plants13081125/s1, Table S1: Temperature values during saffron cultivation; Figure S1: Appearance of saffron plants on the different dates when samples were collected for enzymatic analysis. Scale bars = 1 cm; Figure S2: Summary diagram showing the analyzed characters and corresponding sampling dates across the different experiments conducted in this study; Figure S3: Estimated number of cells of the daughter corm from January to April taking into account the measured cell size, and the volume of the daughter corm. Different letters indicate significant differences between dates (*p* < 0.05); Figure S4: Percentage of total metabolizable soluble sugars [SS] (A) and starch (B) on the different organs of saffron (daughter corm, DC; mother corm, MC and leaves, LFs) during the vegetative period. Values are averages of three independent samples. In each graph, different letters indicate significant differences (*p* < 0.05) between organs for each date.
