# Supplementary material for: Understanding the Saffron Corm Development—Insights into Histological and Metabolic Aspects
Source: Plants (Basel). 2024 Apr 17;13(8):1125. doi: 10.3390/plants13081125 (PMC11055066; doi:10.3390/plants13081125)
Supplement: Supplementary file 1 [file plants-13-01125-s001.zip › Figure S1.pdf]

Figure S1. Appearance of saffron plants on the different dates when samples were collected for enzymatic analysis. Scale bars = 1cm

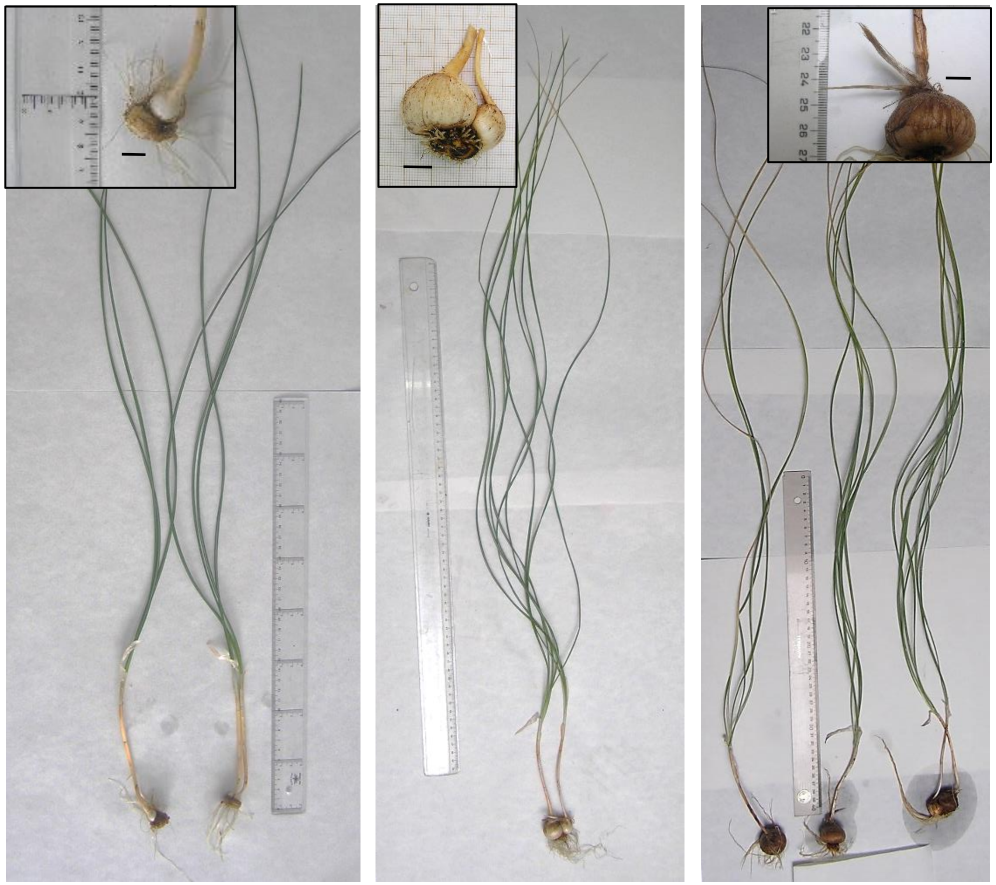

January 17th March 21st April 17th
