# Supplementary material for: Understanding the Saffron Corm Development—Insights into Histological and Metabolic Aspects
Source: Plants (Basel). 2024 Apr 17;13(8):1125. doi: 10.3390/plants13081125 (PMC11055066; doi:10.3390/plants13081125)
Supplement: Supplementary file 1 [file plants-13-01125-s001.zip › Figure S2.pdf]

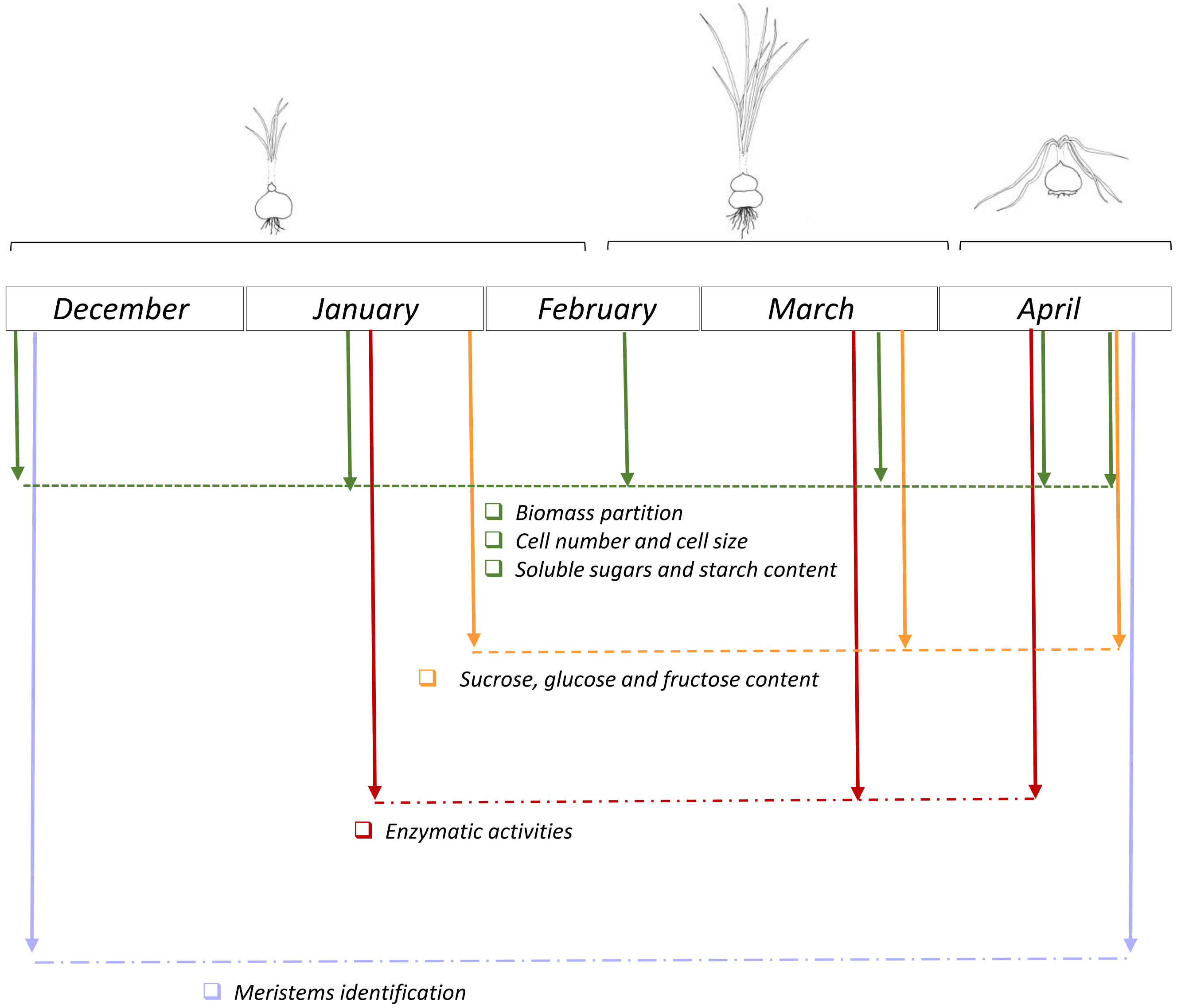

Figure S2. Summary diagram showing the analyzed characters and corresponding sampling dates across the different experiments conducted in this study.
