# Supplementary material for: Understanding the Saffron Corm Development—Insights into Histological and Metabolic Aspects
Source: Plants (Basel). 2024 Apr 17;13(8):1125. doi: 10.3390/plants13081125 (PMC11055066; doi:10.3390/plants13081125)

Figure S3. Estimated number of cells of the daughter corm from January to April taking into account the measured cell size, and the volume of the daughter corm. Different letters indicate significant differences between dates (P<0.05).

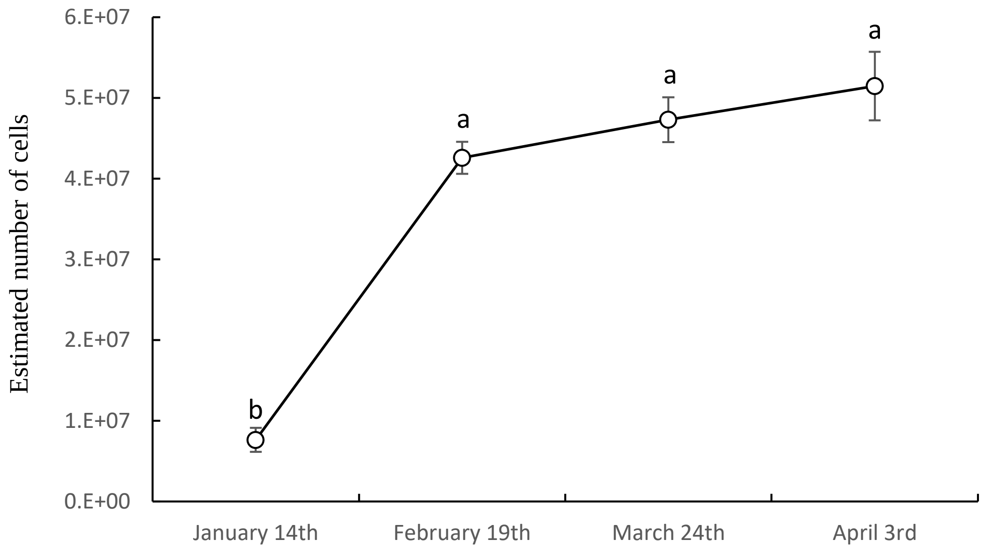

Supplement: Supplementary file 1 [file plants-13-01125-s001.zip › Figure S3.pdf]
