# Supplementary material for: Understanding the Saffron Corm Development—Insights into Histological and Metabolic Aspects
Source: Plants (Basel). 2024 Apr 17;13(8):1125. doi: 10.3390/plants13081125 (PMC11055066; doi:10.3390/plants13081125)

Figure S4. Percentage of total metabolizable soluble sugars [SS] (A) and starch (B) on the different organs of saffron (daughter corm, DC; mother corm, MC and leaves, LFs) during the vegetative period. Values are averages of three independent samples. Within each graph, different letters indicate significant differences (P < 0.05) between organs for each date.

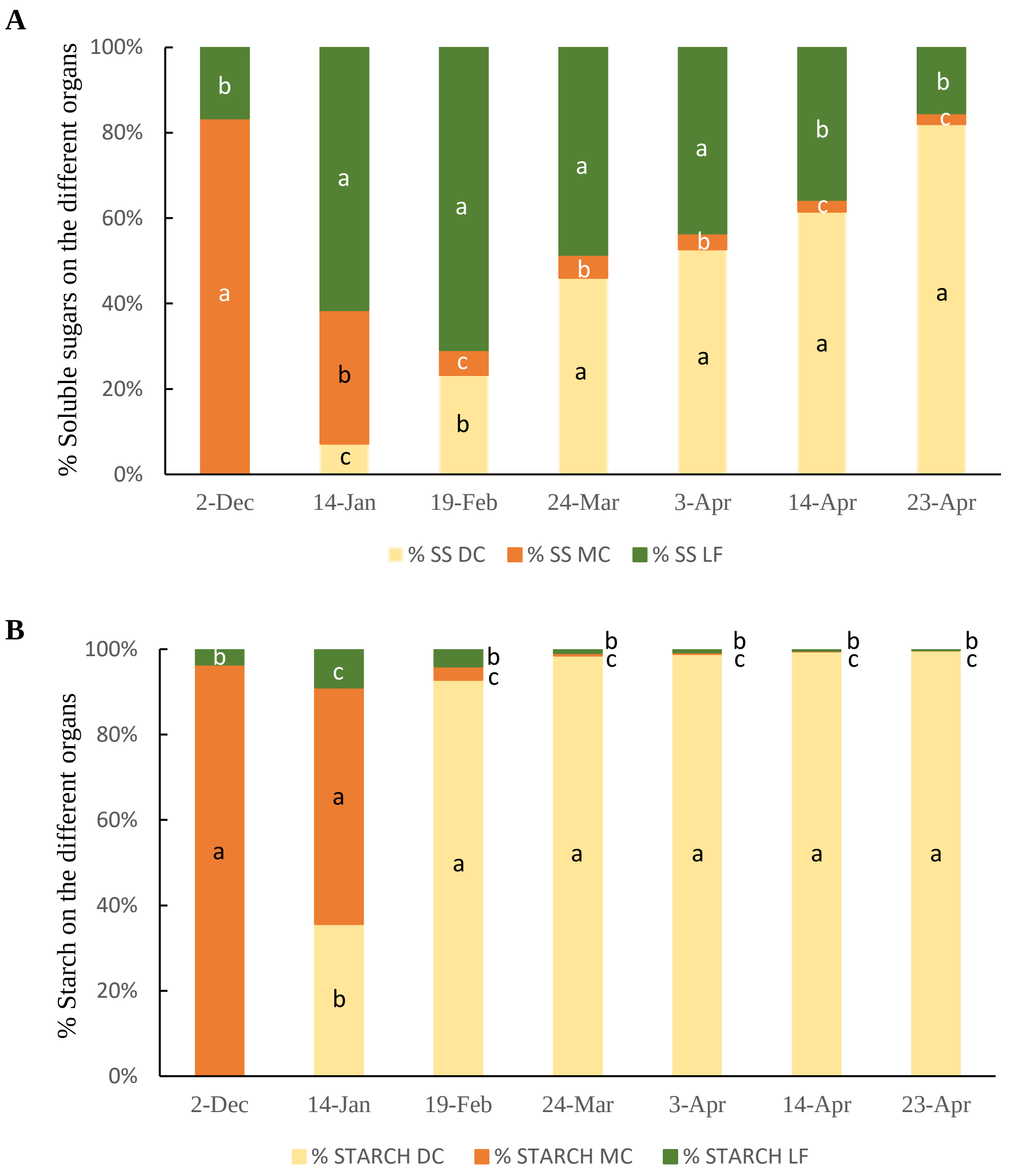

Supplement: Supplementary file 1 [file plants-13-01125-s001.zip › Figure S4.pdf]
