# Supplementary material for: Understanding the Saffron Corm Development—Insights into Histological and Metabolic Aspects
Source: Plants (Basel). 2024 Apr 17;13(8):1125. doi: 10.3390/plants13081125 (PMC11055066; doi:10.3390/plants13081125)
Supplement: Supplementary file 1 [file plants-13-01125-s001.zip › Table S1.pdf]

Supplemental Table S1. Temperature values during saffron cultivation

A) Temperature values in the experimental field

	Mean Temperature (°C)	Mean Min. Temperature (°C)	Mean Max. Temperature (°C)
October	22.4	14.5	27.8
November	15.8	7.2	21.1
December	12.4	3.7	17.5
January	14.1	6.0	17.8
February	14.2	6.1	18.3
March	14.8	6.3	20.0
April	18.7	10.7	24.3
May	19.5	12.2	24.6

B) Temperature values in the greenhouse

Date	Mean Temperature (°C)	Mean Min. Temperature (°C)	Mean Max. Temperature (°C)
October	23.2	18.6	29.2
November	19.4	14.4	27.4
December	18.4	13.3	27.4
January	18.8	12.8	28.1
February	20.2	14.4	28.3
March	22.6	15.2	32.6
April	22.1	14.8	29.4
May	23.6	17.1	30.3
